# Supplementary material for: Effect of exercise training on heart rate variability in type 2 diabetes mellitus patients: A systematic review and meta-analysis
Source: PLoS One. 2021 May 17;16(5):e0251863. doi: 10.1371/journal.pone.0251863 (PMC8128270; doi:10.1371/journal.pone.0251863)
Supplement: S1 Appendix — (DOCX) [file pone.0251863.s002.docx]

**Electronic Supplementary Material Appendix S2**

**Quality of studies – Grids**
