# Supplementary material for: Effect of exercise training on heart rate variability in type 2 diabetes mellitus patients: A systematic review and meta-analysis
Source: PLoS One. 2021 May 17;16(5):e0251863. doi: 10.1371/journal.pone.0251863 (PMC8128270; doi:10.1371/journal.pone.0251863)
Supplement: S2 Appendix — (PDF) [file pone.0251863.s003.pdf]

PEDro scale

1.	eligibility criteria were specified	no ❑ yes ❑ where:
2.	subjects were randomly allocated to groups (in a crossover study, subjects were randomly allocated an order in which treatments were received)	no ❑ yes ❑ where:
3.	allocation was concealed	no ❑ yes ❑ where:
4.	the groups were similar at baseline regarding the most important prognostic indicators	no ❑ yes ❑ where:
5.	there was blinding of all subjects	no ❑ yes ❑ where:
6.	there was blinding of all therapists who administered the therapy	no ❑ yes ❑ where:
7.	there was blinding of all assessors who measured at least one key outcome	no ❑ yes ❑ where:
8.	measures of at least one key outcome were obtained from more than 85% of the subjects initially allocated to groups	no ❑ yes ❑ where:
9.	all subjects for whom outcome measures were available received the treatment or control condition as allocated or, where this was not the case, data for at least one key outcome was analysed by "intention to treat"	no ❑ yes ❑ where:
10.	the results of between-group statistical comparisons are reported for at least one key outcome	no ❑ yes ❑ where:
11.	the study provides both point measures and measures of variability for at least one key outcome	no ❑ yes ❑ where:

The PEDro scale is based on the Delphi list developed by Verhagen and colleagues at the Department of Epidemiology, University of Maastricht (*Verhagen AP et al (1998). The Delphi list: a criteria list for quality assessment of randomised clinical trials for conducting systematic reviews developed by Delphi consensus. Journal of Clinical Epidemiology, 51(12):1235-41*). The list is based on "expert consensus" not, for the most part, on empirical data. Two additional items not on the Delphi list (PEDro scale items 8 and 10) have been included in the PEDro scale. As more empirical data comes to hand it may become possible to "weight" scale items so that the PEDro score reflects the importance of individual scale items.

The purpose of the PEDro scale is to help the users of the PEDro database rapidly identify which of the known or suspected randomised clinical trials (ie RCTs or CCTs) archived on the PEDro database are likely to be internally valid (criteria 2-9), and could have sufficient statistical information to make their results interpretable (criteria 10-11). An additional criterion (criterion 1) that relates to the external validity (or "generalisability" or "applicability" of the trial) has been retained so that the Delphi list is complete, but this criterion will not be used to calculate the PEDro score reported on the PEDro web site.

The PEDro scale should not be used as a measure of the "validity" of a study's conclusions. In particular, we caution users of the PEDro scale that studies which show significant treatment effects and which score highly on the PEDro scale do not necessarily provide evidence that the treatment is clinically useful. Additional considerations include whether the treatment effect was big enough to be clinically worthwhile, whether the positive effects of the treatment outweigh its negative effects, and the cost-effectiveness of the treatment. The scale should not be used to compare the "quality" of trials performed in different areas of therapy, primarily because it is not possible to satisfy all scale items in some areas of physiotherapy practice.

Last amended June 21st, 1999

Notes on administration of the PEDro scale:

All criteria	**Points are only awarded when a criterion is clearly satisfied**. If on a literal reading of the trial report it is possible that a criterion was not satisfied, a point should not be awarded for that criterion.
Criterion 1	This criterion is satisfied if the report describes the source of subjects and a list of criteria used to determine who was eligible to participate in the study.
Criterion 2	A study is considered to have used random allocation if the report states that allocation was random. The precise method of randomisation need not be specified. Procedures such as coin-tossing and dice-rolling should be considered random. Quasi-randomisation allocation procedures such as allocation by hospital record number or birth date, or alternation, do not satisfy this criterion.
Criterion 3	*Concealed allocation* means that the person who determined if a subject was eligible for inclusion in the trial was unaware, when this decision was made, of which group the subject would be allocated to. A point is awarded for this criteria, even if it is not stated that allocation was concealed, when the report states that allocation was by sealed opaque envelopes or that allocation involved contacting the holder of the allocation schedule who was "off-site".
Criterion 4	At a minimum, in studies of therapeutic interventions, the report must describe at least one measure of the severity of the condition being treated and at least one (different) key outcome measure at baseline. The rater must be satisfied that the groups' outcomes would not be expected to differ, on the basis of baseline differences in prognostic variables alone, by a clinically significant amount. This criterion is satisfied even if only baseline data of study completers are presented.
Criteria 4, 7-11	*Key outcomes* are those outcomes which provide the primary measure of the effectiveness (or lack of effectiveness) of the therapy. In most studies, more than one variable is used as an outcome measure.
Criterion 5-7	*Blinding* means the person in question (subject, therapist or assessor) did not know which group the subject had been allocated to. In addition, subjects and therapists are only considered to be "blind" if it could be expected that they would have been unable to distinguish between the treatments applied to different groups. In trials in which key outcomes are self-reported (eg, visual analogue scale, pain diary), the assessor is considered to be blind if the subject was blind.
Criterion 8	This criterion is only satisfied if the report explicitly states *both* the number of subjects initially allocated to groups *and* the number of subjects from whom key outcome measures were obtained. In trials in which outcomes are measured at several points in time, a key outcome must have been measured in more than 85% of subjects at one of those points in time.
Criterion 9	An *intention to treat* analysis means that, where subjects did not receive treatment (or the control condition) as allocated, and where measures of outcomes were available, the analysis was performed as if subjects received the treatment (or control condition) they were allocated to. This criterion is satisfied, even if there is no mention of analysis by intention to treat, if the report explicitly states that all subjects received treatment or control conditions as allocated.
Criterion 10	A *between-group* statistical comparison involves statistical comparison of one group with another. Depending on the design of the study, this may involve comparison of two or more treatments, or comparison of treatment with a control condition. The analysis may be a simple comparison of outcomes measured after the treatment was administered, or a comparison of the change in one group with the change in another (when a factorial analysis of variance has been used to analyse the data, the latter is often reported as a group × time interaction). The comparison may be in the form hypothesis testing (which provides a "p" value, describing the probability that the groups differed only by chance) or in the form of an estimate (for example, the mean or median difference, or a difference in proportions, or number needed to treat, or a relative risk or hazard ratio) and its confidence interval.
Criterion 11	A *point measure* is a measure of the size of the treatment effect. The treatment effect may be described as a difference in group outcomes, or as the outcome in (each of) all groups. *Measures of variability* include standard deviations, standard errors, confidence intervals, interquartile ranges (or other quantile ranges), and ranges. Point measures and/or measures of variability may be provided graphically (for example, SDs may be given as error bars in a Figure) as long as it is clear what is being graphed (for example, as long as it is clear whether error bars represent SDs or SEs). Where outcomes are categorical, this criterion is considered to have been met if the number of subjects in each category is given for each group.
