# Supplementary figures and images for: Effect of exercise training on heart rate variability in type 2 diabetes mellitus patients: A systematic review and meta-analysis
Source: PLoS One. 2021 May 17;16(5):e0251863. doi: 10.1371/journal.pone.0251863 (PMC8128270; doi:10.1371/journal.pone.0251863)

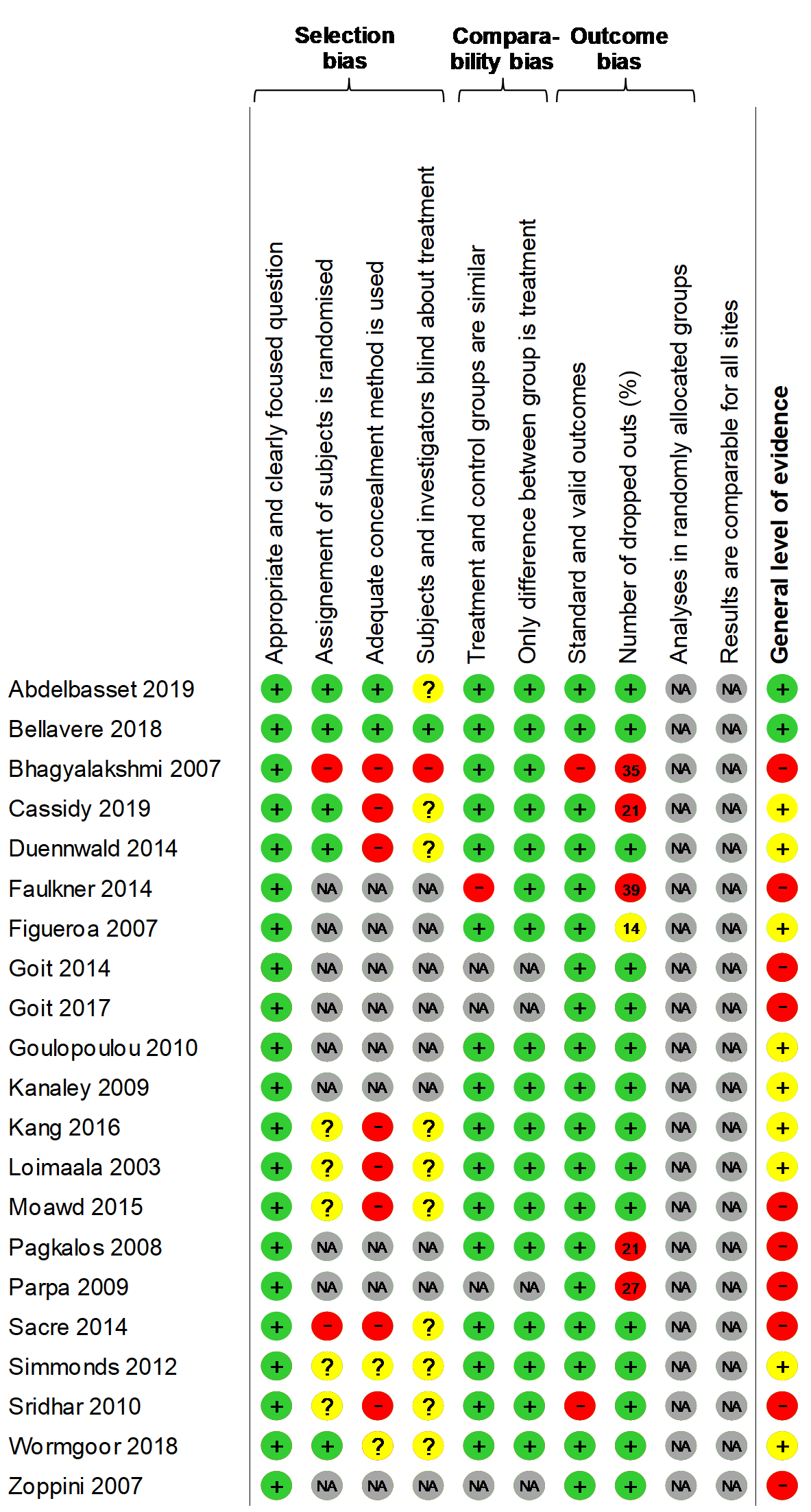

Supplement: S1 Fig — For each item, criteria fulfilled: No: -, Yes: +, Unclear:?, Not applicable: NA. (TIF) [file pone.0251863.s004.tif]

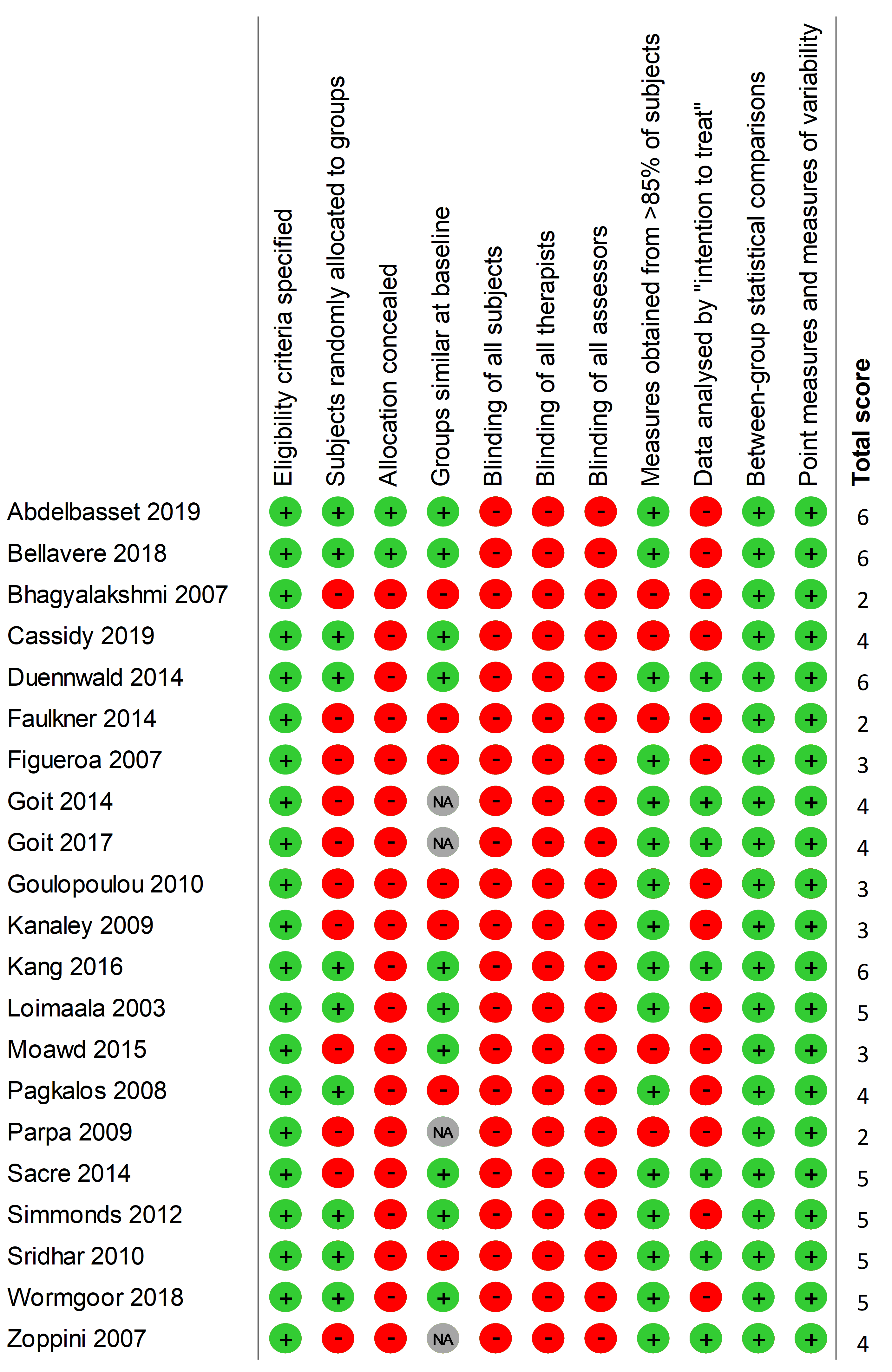

Supplement: S2 Fig — (TIF) [file pone.0251863.s005.tif]

# rr\_ms in T2D after exercise compared to baseline and in controls without exercise

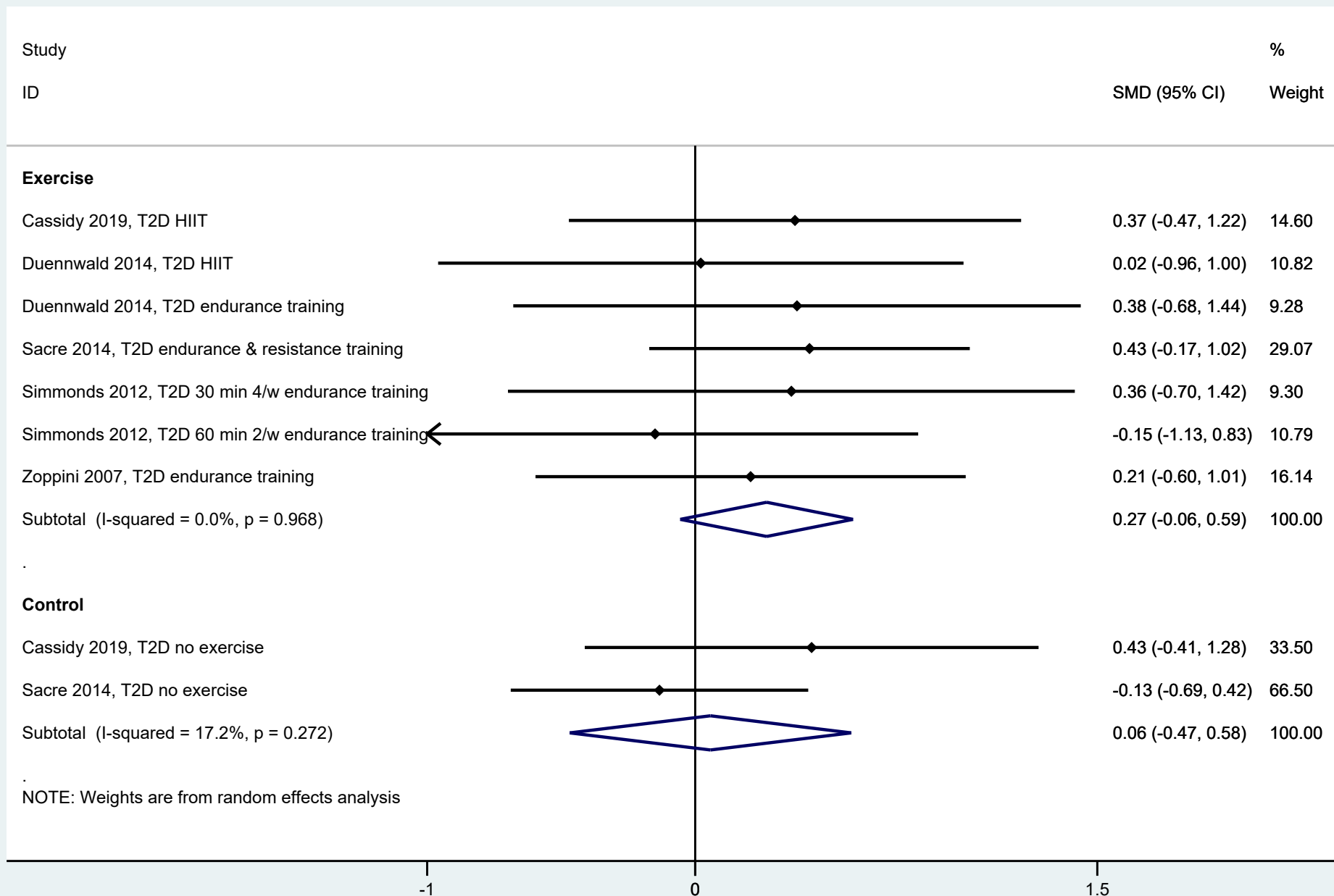

Supplement: S3 Fig — (PDF) [file pone.0251863.s006.pdf]

# SDNN in T2D after exercise compared to baseline and in controls without exercise

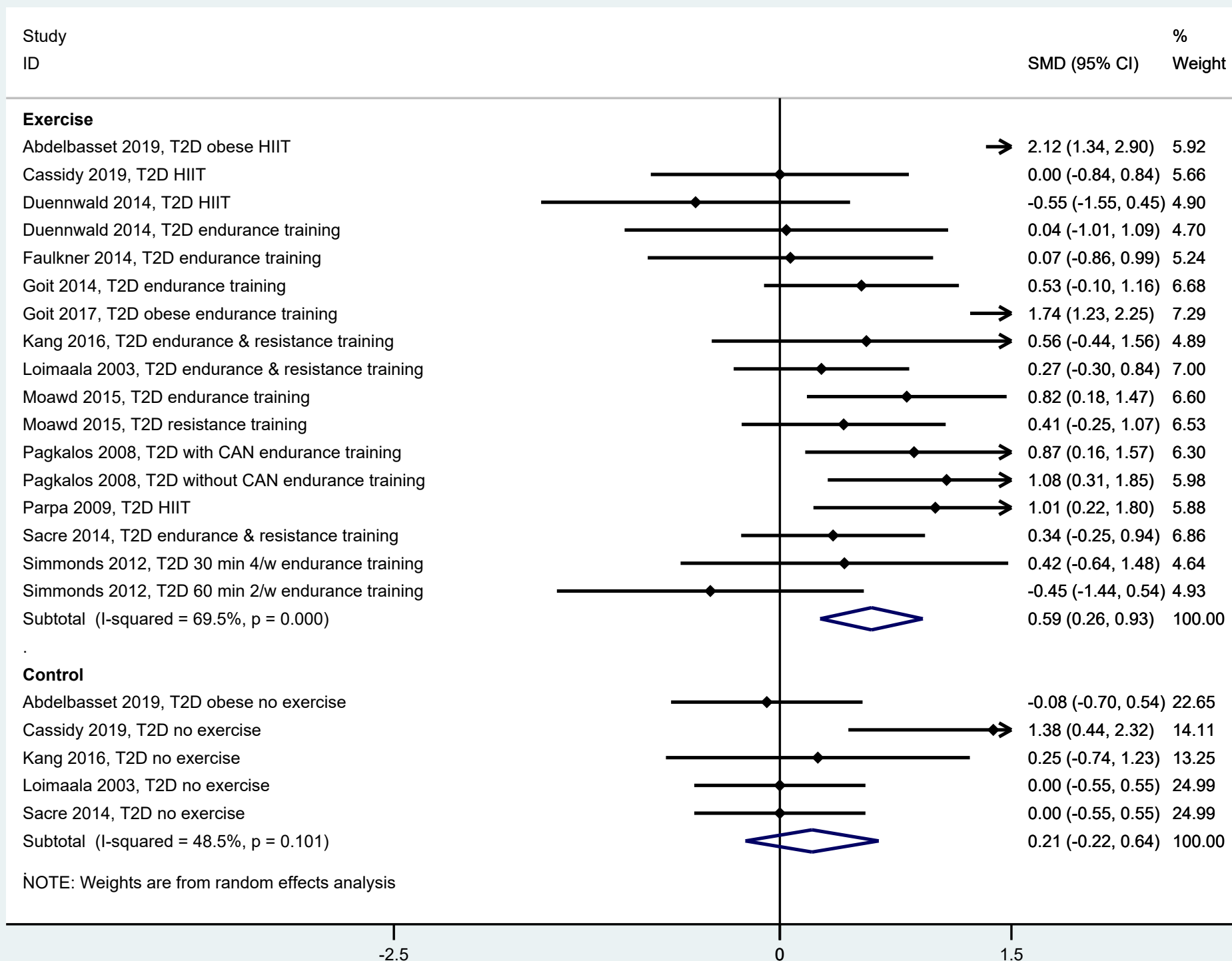

Supplement: S4 Fig — (PDF) [file pone.0251863.s007.pdf]

# rMSSD in T2D after exercise compared to baseline and in controls without exercise

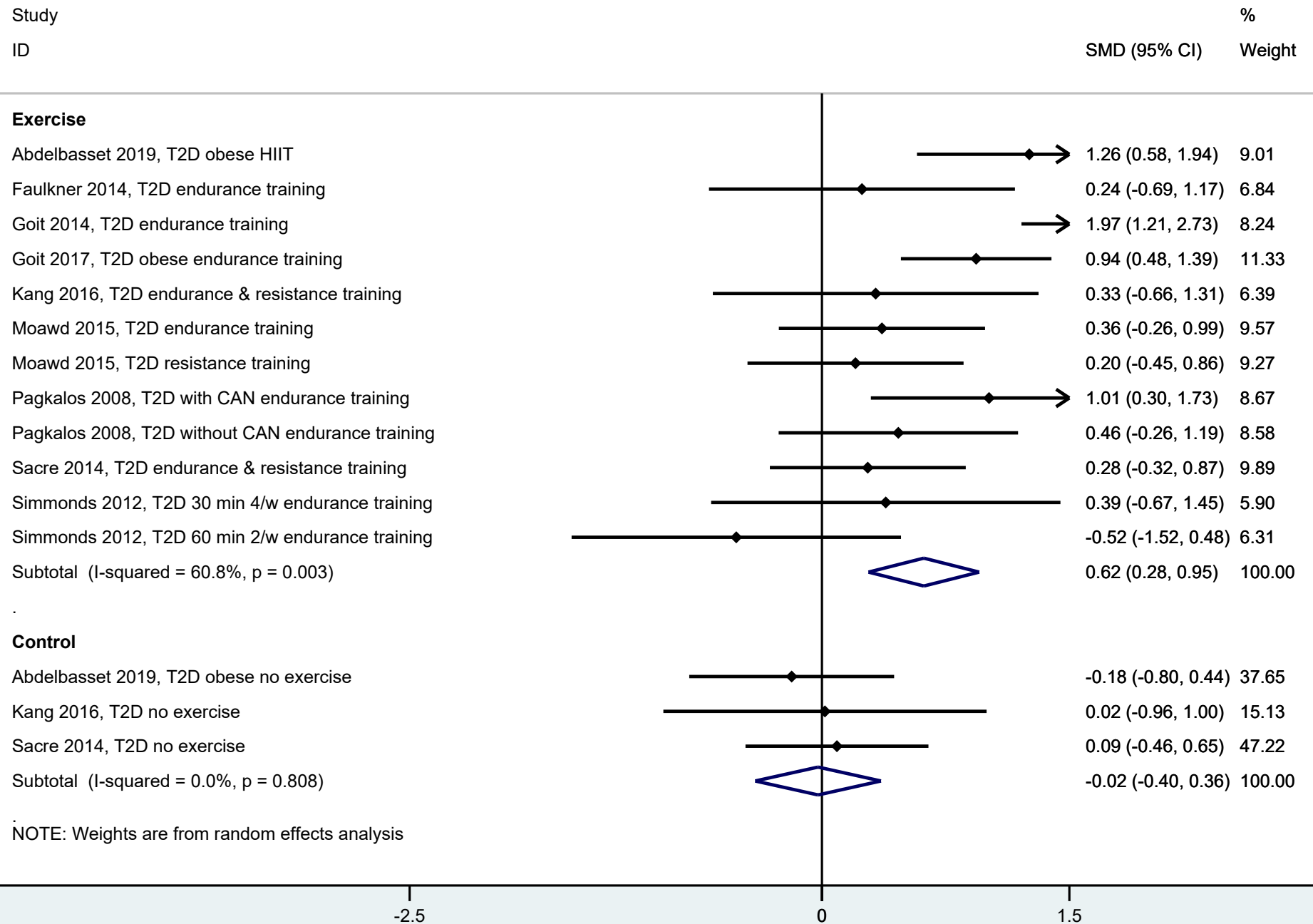

Supplement: S5 Fig — (PDF) [file pone.0251863.s008.pdf]

# pNN50 in T2D after exercise compared to baseline and in controls without exercise

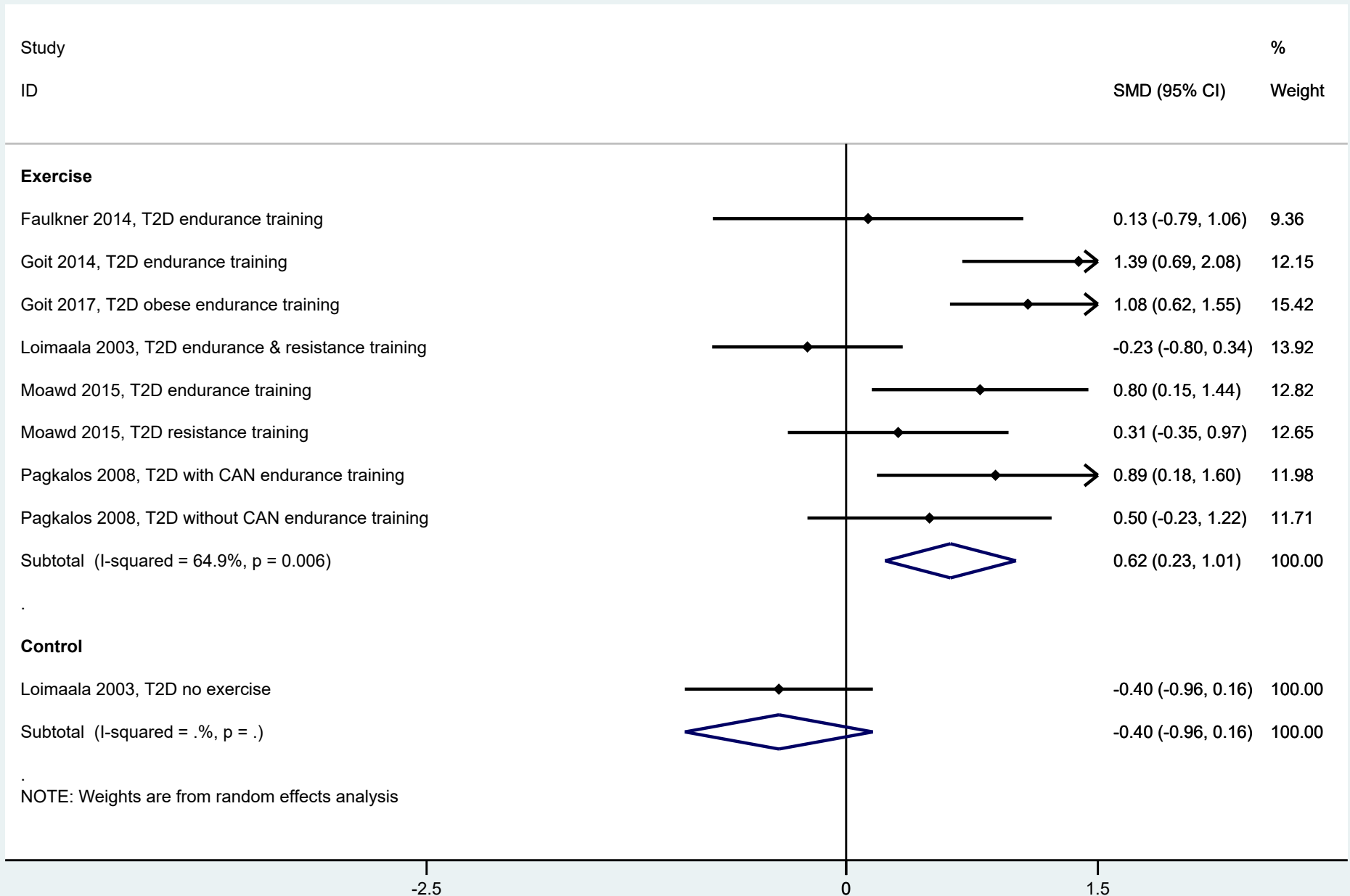

Supplement: S6 Fig — (PDF) [file pone.0251863.s009.pdf]

# Total Power in T2D after exercise compared to baseline and in controls without exercise

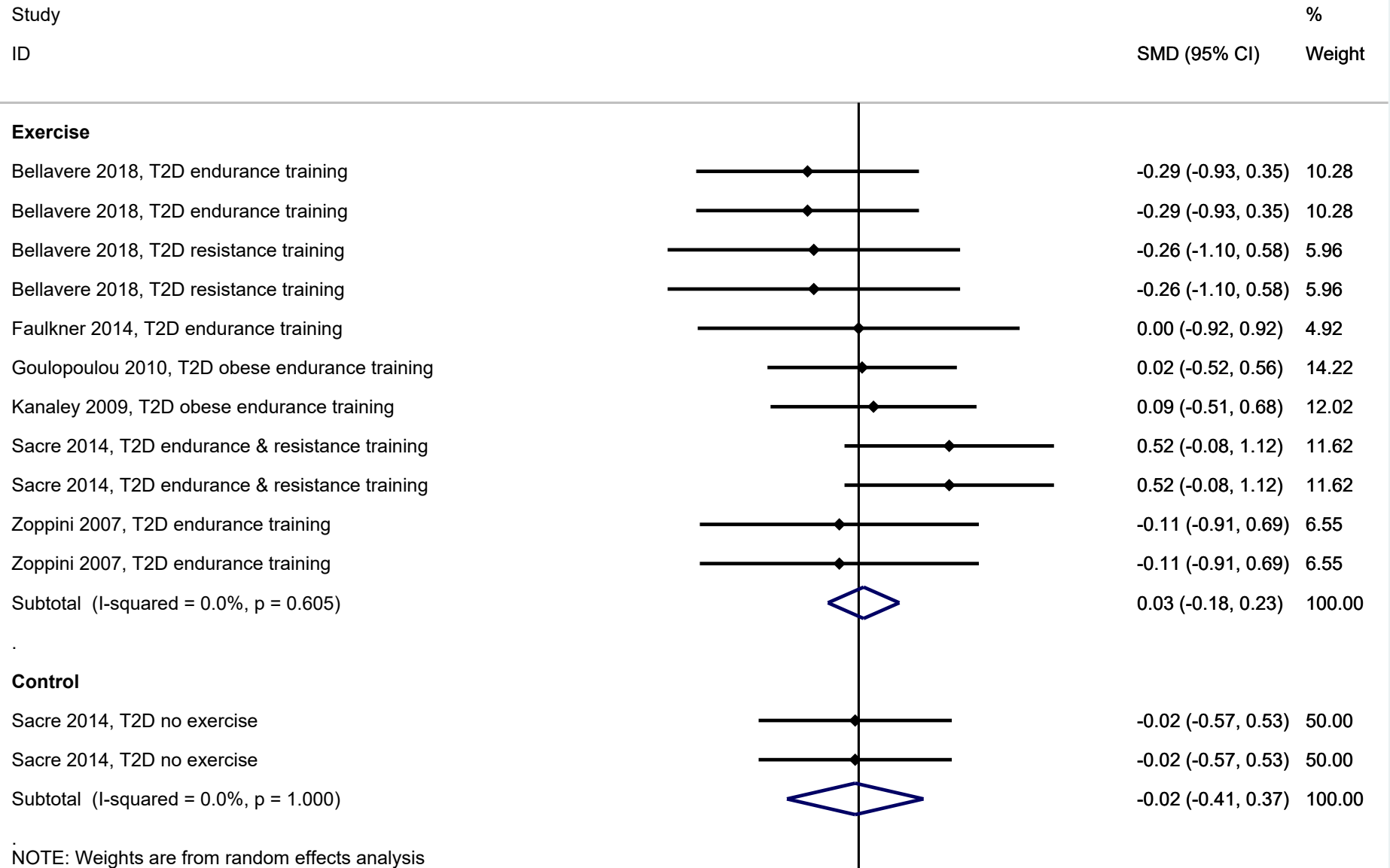

Supplement: S7 Fig — (PDF) [file pone.0251863.s010.pdf]

# LF in T2D after exercise compared to baseline and in controls without exercise

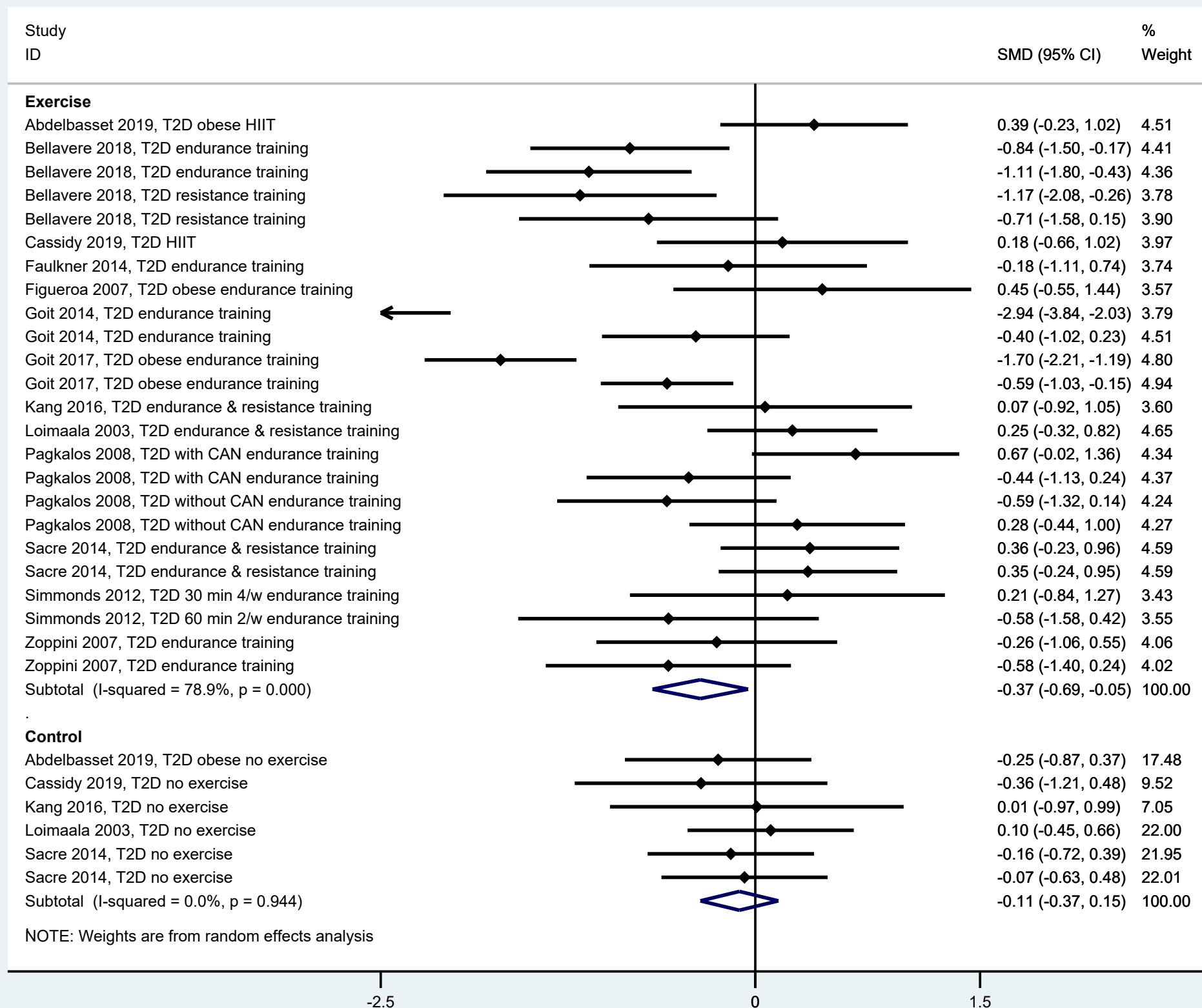

Supplement: S8 Fig — (PDF) [file pone.0251863.s011.pdf]

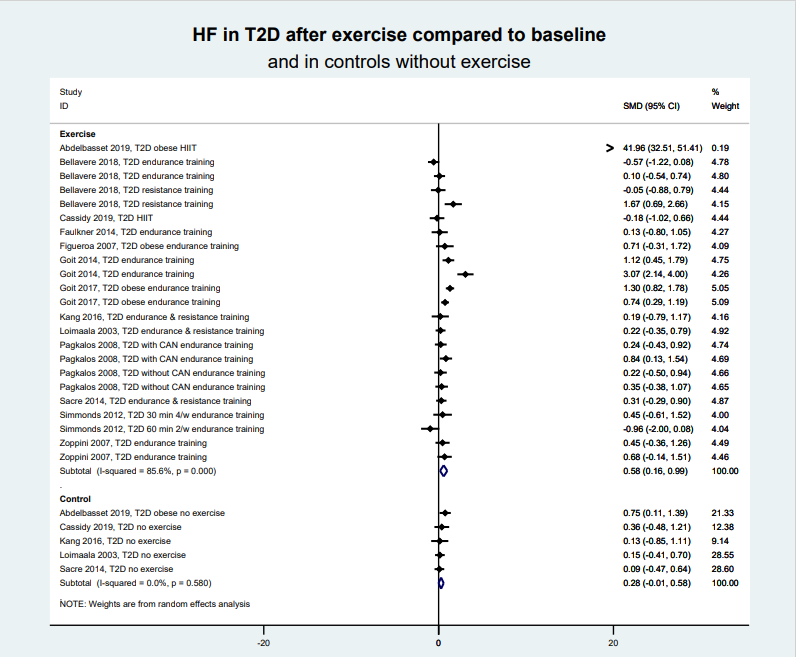

Supplement: S9 Fig — (PNG) [file pone.0251863.s012.png]

# LF/HF in T2D after exercise compared to baseline and in controls without exercise

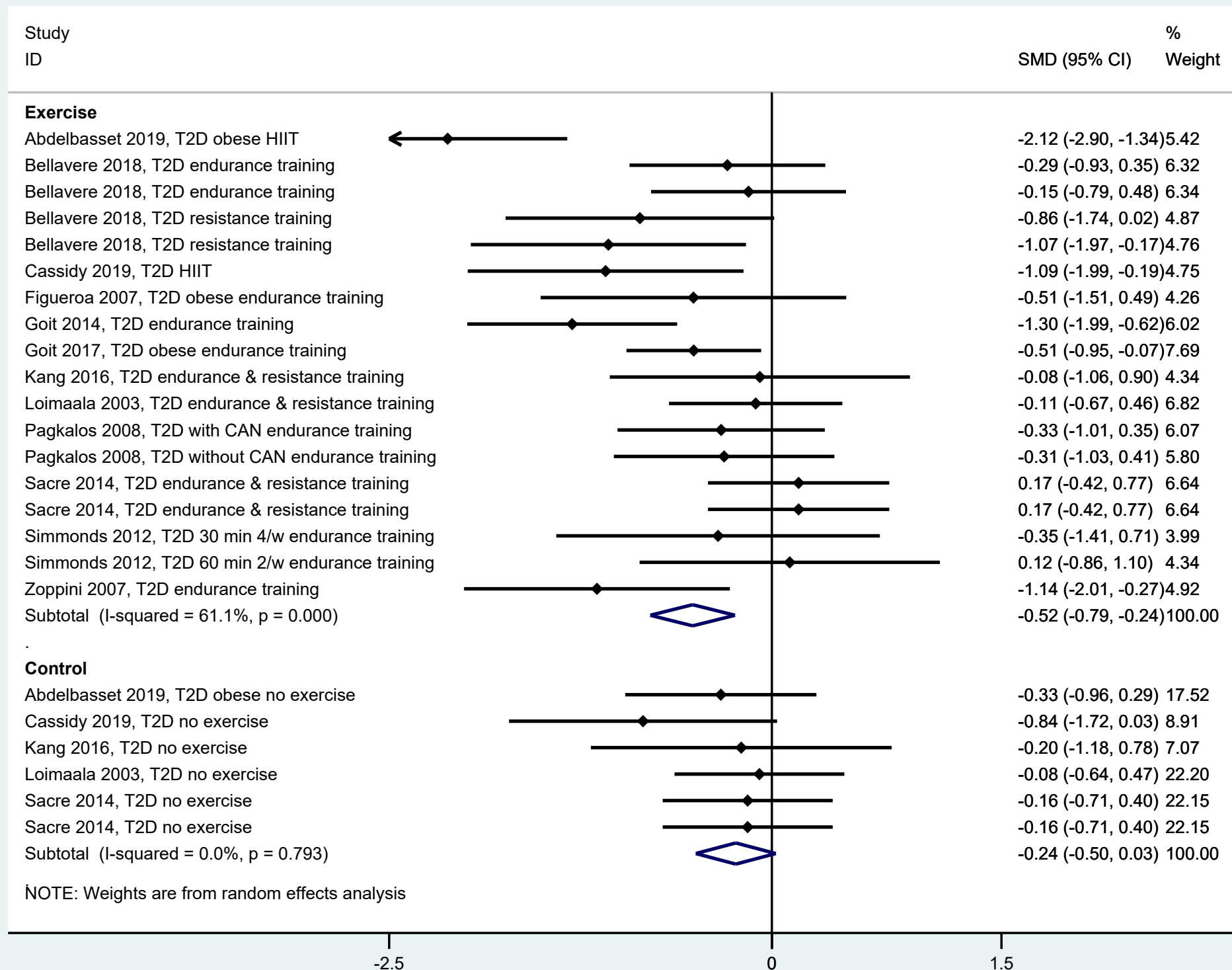

Supplement: S10 Fig — (PDF) [file pone.0251863.s013.pdf]

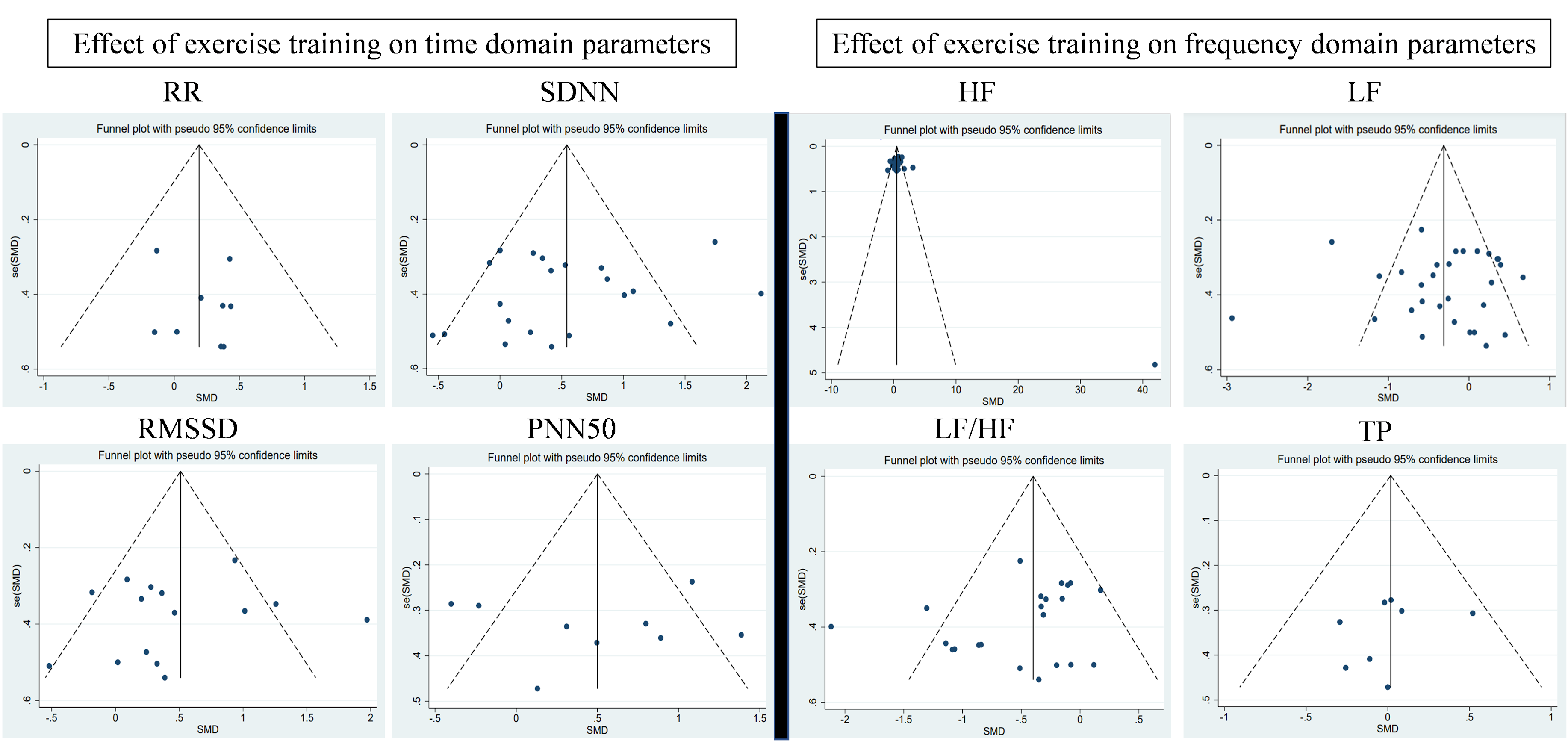

Supplement: S11 Fig — (TIF) [file pone.0251863.s014.tif]

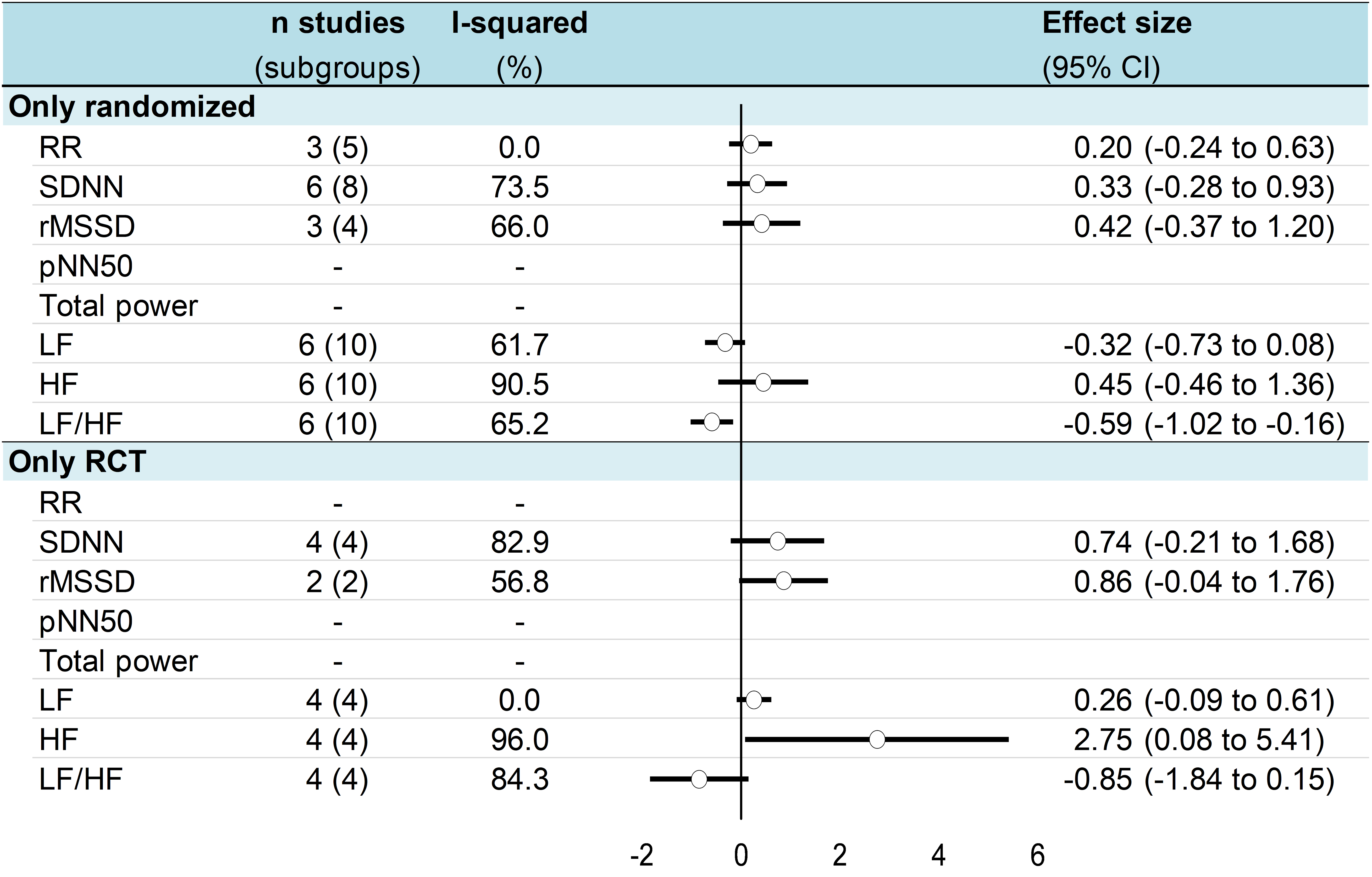

Supplement: S12 Fig — (TIF) [file pone.0251863.s015.tif]
